# Insights Into Transcriptome Profiles Associated With Wooden Breast Myopathy in Broilers Slaughtered at the Age of 6 or 7 Weeks

**DOI:** 10.3389/fphys.2021.691194

**Published:** 2021-06-28

**Authors:** Yuwares Malila, Tanaporn Uengwetwanit, Krittaporn V. Thanatsang, Sopacha Arayamethakorn, Yanee Srimarut, Massimiliano Petracci, Francesca Soglia, Wanilada Rungrassamee, Wonnop Visessanguan

**Affiliations:** ^1^National Center for Genetic Engineering and Biotechnology (BIOTEC), Thailand Science Park, Pathum Thani, Thailand; ^2^Department of Agricultural and Food Sciences, Alma Mater Studiorum, University of Bologna, Cesena, Italy

**Keywords:** broiler chicken, wooden breast, transcriptome profile, droplet digital polymerase chain reaction, focal adhesion

## Abstract

Transcriptomes associated with wooden breast (WB) were characterized in broilers at two different market ages. Breasts (*Pectoralis major*) were collected, 20-min postmortem, from male Ross 308 broilers slaughtered at 6 and 7 weeks of age. The breasts were classified as “non-WB” or “WB” based on palpation hardness scoring (non-WB = no abnormal hardness, WB = consistently hardened). Total RNA was isolated from 16 samples (*n* = 3 for 6 week non-WB, *n* = 3 for 6 week WB; *n* = 5 for 7 week non-WB, *n* = 5 for 7 week WB). Transcriptome was profiled using a chicken gene expression microarray with one-color hybridization technique, and compared between non-WB and WB samples of the same age. Among 6 week broilers, 910 transcripts were differentially expressed (DE) (false discovery rate, FDR < 0.05). Pathway analysis underlined metabolisms of glucose and lipids along with gap junctions, tight junction, and focal adhesion (FA) signaling as the top enriched pathways. For the 7 week broilers, 1,195 transcripts were identified (FDR < 0.05) with regulation of actin cytoskeleton, mitogen-activated protein kinase (MAPK) signaling, protein processing in endoplasmic reticulum and FA signaling highlighted as the enriched affected pathways. Absolute transcript levels of eight genes (actinin-1 – *ACTN1*, integrin-linked kinase – *ILK*, integrin subunit alpha 8 – *ITGA8*, integrin subunit beta 5 – *ITGB5*, protein tyrosine kinase 2 – *PTK2*, paxillin – *PXN*, talin 1 – *TLN1*, and vinculin – *VCL*) of FA signaling pathway were further elucidated using a droplet digital polymerase chain reaction. The results indicated that, in 6 week broilers, *ITGA8* abundance in WB was greater than that of non-WB samples (*p* < 0.05). Concerning 7 week broilers, greater absolute levels of *ACTN1*, *ILK*, *ITGA8*, and *TLN1*, accompanied with a reduced *ITGB5* were found in WB compared with non-WB (*p* < 0.05). Transcriptional modification of FA signaling underlined the potential of disrupted cell-cell communication that may incite aberrant molecular events in association with development of WB myopathy.

## Introduction

Wooden breast (WB) abnormality, one of the emerging growth-related myopathies affecting chicken pectoral muscle, is characterized by hardened ridges extending from the cranial to the caudal regions of the breast (Sihvo et al., 2014). The abnormal breasts often exhibit pale color, surface hemorrhagic lesions, and clear viscous exudate on the surface (Barbut, 2019). Identified shortly after white striping (WS) abnormality, WB has abruptly raised a wide concern in the broiler industry worldwide (Sihvo et al., 2014; Velleman and Clark, 2015; de Carvalho et al., 2020; Xing et al., 2020) as it exerts remarkable adverse impacts on technological properties, including water holding capacity and texture, of chicken breasts (Chatterjee et al., 2016; Soglia et al., 2016; Tasoniero et al., 2016; Tijare et al., 2016; Cai et al., 2018; Malila et al., 2018; Madruga et al., 2019). Hence, it has caused tremendous economic losses. In United States alone, it was estimated that the growth-related myopathies had costed approximately 70,000 USD losses daily (Zanetti et al., 2018).

The actual etiology and the chronological events underlying development of WB are under extensive investigation. A growing evidence collected from scientific reports have pointed out the association between WB myopathy with artificial selection for rapid growth rate, enlarged muscle mass, and accelerated metabolism (Malila et al., 2018; Barbut, 2019; Petracci et al., 2019). The disproportionate muscles outgrew their supportive systems, particularly vascularization and oxygenation, leading to a series of molecular and ultrastructural disturbances that negatively affected myocellular integrity (Papah et al., 2018; Papah and Abasht, 2019; Maharjan et al., 2020; Abasht et al., 2021). Signs of phlebitis along with muscle damage and regeneration were observed as soon as 14 days of age in broilers (Papah et al., 2017; Radaelli et al., 2017). Damaged muscle fibers were replaced by collagen and adipose tissue and exhibited altered sarcomeric structure and functions (Papah et al., 2017; Velleman et al., 2017; Liu et al., 2020). Metabolic processes, ion homeostasis, as well as oxidative stress response mechanisms can be profoundly disrupted (Mutryn et al., 2015; Abasht et al., 2016; Kang et al., 2020). Soglia et al. (2020) recently demonstrated an increase in vimentin (VIM) and desmin (DES) in WB muscles compared with those of normal counterparts, suggesting the occurrence of intensive regenerative processes within these muscles. Muscle regeneration requires the donation of nuclei from satellite cells, the myogenic stem cells required in post-hatch skeletal muscle growth and regeneration (Velleman, 2015). In agreement with the on-going regenerative process in the presence of WB myopathy, numbers of total nuclei and mitotically active satellite cells were increased in the 43 day broilers compared with those of non-WB (Meloche et al., 2018). However, the mitotic activity of the satellite cell population was significantly altered in WB broiler, suggesting the compromised reparative capacity of the satellite cells in the abnormal birds (Ferreira et al., 2020).

Previous studies demonstrated the effects of age and hypertrophy on increasing the severity of muscle degeneration (Radaelli et al., 2017) and on the losses of satellite cells at both quantitative and functional characteristics, hence impairing muscle regeneration (Daughtry et al., 2017) in broilers. As described by Domingues-Faria et al. (2016), when skeletal muscle undergoes damage, pro-inflammatory cytokines recruit immune cells to the lesion site thus promoting migration and proliferation of satellite cells. Neutrophils take parts in the lysis of damaged cells as well as in phagocytosis to remove cell debris through apoptosis. The pro-inflammatory macrophages are then converted into anti-inflammatory phenotype to initiate differentiation of satellite cells, hence muscle repair. Overall, the repair process requires a well-regulated crosstalk between the muscle cells and the immune cells. In this regard, it is worth mentioning that, although affected by WB at a similar extent, broilers slaughtered at different ages might exhibit different molecular responses. Based on our previous study (Malila et al., 2018), the greater numbers of WB cases were found in the commercial broilers slaughtered at the age of 7 weeks in comparison to those slaughtered at 6 weeks. The increased WB severity was significantly related with the increased proportion of breast mass relative to carcass weight which was observed more in the group of 7-week-old broilers (Malila et al., 2018). Thus, performing further investigations may improve the knowledge relating to key biological processes associated with the development of WB. Here, the transcriptional profiles of non-WB and WB samples collected from commercial broilers at 6 and 7 weeks of age were assessed in order to investigate the progression of the muscular alterations occurring when the slaughter age was postponed.

## Materials and Methods

### Samples and Sample Collection

The breast samples used in this study were collected from carcasses of Ross 308 broilers, males, reared in a commercial farm until the age of 6 weeks (6wk) or 7 weeks (7wk). All birds were slaughtered at a local industrial-scale slaughterhouse. After the scalding step, the carcasses were randomly collected from the processing line, the breasts were dissected, and classified as “non-WB” or “WB” based on the presence of hardened bulge ridges assessed by palpation (Sihvo et al., 2017). The WB samples used in this study could be classified as “severe WB” (>75% of the breast being extremely rigid and with diffuse coverage) (Sihvo et al., 2017; Petracci et al., 2019). A sample was excised from the cranial portion (approximately 1 cm deep from the ventral surface) of the muscle, cut into 1 cm^3^ cubes, snap frozen in liquid nitrogen and stored at −80°C until RNA isolation.

Among the samples collected from 6 and 7-week-old birds, three and five samples were identified as WB, respectively. It is worth noting that all WB samples showed macroscopic characteristics of WS. The equal numbers of 6 and 7 week breast samples exhibiting no abnormal hardness were randomly selected to represent the non-WB group in this study. Hence, 16 selected samples were classified into four groups based on age and abnormality, i.e., 6wk; non-WB (*n* = 3), 6wk; WB (*n* = 3), 7wk; non-WB (*n* = 5), and 7wk; WB (*n* = 5).

### Total RNA Isolation

Total RNA was isolated using TRIzol^TM^ Reagent (Invitrogen), subsequently treated with DNase I (Thermo Scientific, Inc.) and re-purified using GeneJET RNA Cleanup and Concentration Micro Kit (Thermo Scientific, Inc.). The samples with an RNA Integrity Number exceeding 7.0 were analyzed by microarray hybridization at Molecular Genomics Pte., Ltd., (Singapore), quantitative real-time polymerase chain reaction (qPCR), and droplet digital PCR (ddPCR).

### Microarray Hybridization and Data Analysis

The Agilent SurePrint G3 Custom GE 8 × 60 K chicken gene expression microarray (Agilent Technologies, Inc.) used in this study was designed based on the National Center for Biotechnology Information (NCBI) *Gallus gallus* Annotation Release 103.

One-color microarray hybridization was conducted as described in Malila et al. (2020). Briefly, total RNA was reverse transcribed into cDNA using an oligo(dT) as a primer. The cDNA was *in vitro* transcribed using T7 RNA polymerases to produce cyanine 3-CTP-labeled complementary RNA using a one-color low input quick amp labeling kit (Agilent) following the company’s instruction. The purified cRNA (600 ng) was hybridized onto the Agilent SurePrint array at 65°C for 17 h. The array was subsequently scanned using an Agilent High Resolution Microarray Scanner (C Model, Agilent). The TIFF image was exported and analyzed using an Agilent Feature Extraction Software version V10.7.1.1 (Agilent).

Microarray probe quality control was processed using GeneSpring software. The signal values of probes that were not met the quality control were excluded from the analysis (Malila et al., 2020). The filtered raw data were normalized using quantile normalization (Bolstad, 2017) and combat normalization afterward (Müller et al., 2016; Leek et al., 2017) in R version 3.4.3 (R Core Team, 2017). Log_2_ transformation was applied to normalized data for statistical analysis. Significant differences in the transcript abundance between non-WB vs WB samples belonging to each slaughter age group (6wk and 7wk) were assessed by *t*-test analyses for independent samples. Positive and negative fold change (FC) values represent increased or decreased expression of a particular gene in the WB samples relative to non-WB of the same slaughter age. The differentially expressed (DE) transcripts were identified using combined criteria of false discovery rate (FDR) ≤ 0.05 and absolute fold change (|FC|) ≥ 1.2. All DEGs of each comparison were mapped onto Kyoto Encyclopedia of Genes and Genomes (KEGG) pathway (Kanehisa et al., 2012) to identify possible biological interactions among them.

### Confirmation of Differential Gene Expression

Changes in the expression patterns of the selected genes observed in the current microarray study were confirmed using qPCR (Malila et al., 2015). A total of 30 genes were chosen for confirming the microarray data. Primers (Supplementary Table 1) were designed using Primer-BLAST^1^. Threshold cycle (Ct) was analyzed using BioRad CFX Manager 2.1 software (BioRad). Hypoxanthine-guanine phosphoribosyltransferase (*HPRT*) was assigned as a reference gene as it showed no differences in expression due to WB development (data not shown). For each gene, relative transcript abundance in WB samples relative to non-WB of the same slaughter age was calculated based on 2^–ΔΔCt^ method (Livak and Schmittgen, 2001) and plot against FC analyzed from microarrays.

### Absolute Expression of Genes in Focal Adhesion Signaling Pathway

Absolute expression of eight key genes involved in focal adhesion (FA) signaling pathway was evaluated using an EvaGreen droplet digital polymerase chain reaction (ddPCR) assay of cDNA produced from reverse-transcription reactions. The ddPCR primer sequences are shown in Supplementary Table 1. The 20-μL ddPCR mixture contained 1X EvaGreen^®^ supermix (Bio-Rad Laboratories, Inc., Hercules, CA, United States), 0.25 μM of each forward and reverse primer, and cDNA template. The concentration of the template for each gene was specified in Supplementary Table 1. The template was replaced by an equal volume of nuclease-free water for no template control. The water-in-oil emulsion droplets were generated using a QX100^TM^ droplet generator (Bio-Rad Laboratories, Inc.) according to the manufacturer’s instruction. The droplets were transferred to a 96-well plate, heat sealed and placed into a conventional thermocycler (model T100^TM^, Bio-Rad Laboratories, Inc.). The reaction conditions were 95°C for 5 min; followed by 40 cycles of 95°C for 30 s, 58°C for 1 min, and 4°C for 5 min; and 90°C for 5 min. After the amplification was completed, the plate was transferred to a QX200^TM^ droplet reader (Bio-Rad Laboratories, Inc.) where fluorescent signal intensity of the droplets was measured. The fluorescence amplitude threshold was set under the high amplitude droplet cluster to distinguish positive and negative droplets. The detected droplets were analyzed in copies per 20-μL reaction by QuantaSoft^TM^ software (Bio-Rad Laboratories, Inc.) and divided by the amount of cDNA added to the reaction to obtain the absolute copy numbers per nanogram of template.

The effects of slaughter age and occurrence of WB condition on absolute expression was analyzed based on 2 × 2 factorial analysis of variance. The significant level was set at α = 0.05. The statistical analysis was performed using the R package version 3.4.3.

## Results

### Differential Gene Expression in Chicken Skeletal Muscle Associated With Wooden Breast Abnormality

**FIGURE 1:**
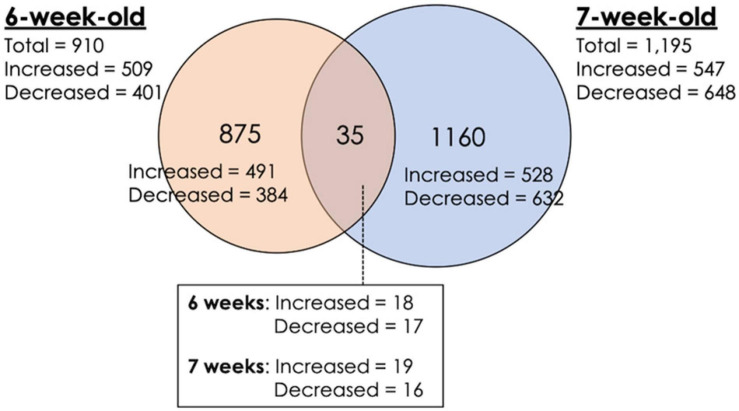
Differential expression of transcripts associated with wooden breast (WB) myopathy in 6-week-old and 7-week-old broilers. Venn diagram depicts number of differentially expressed transcripts obtained from microarray study. Increased expression indicates the greater transcript abundance of the particular genes in WB sample relative to that of non-WB within the same slaughter age, vice versa for decreased expression. The criteria used in identification of differentially expressed transcripts are a combination between false discovery rate ≤ 0.05 and absolute fold change (|FC|) ≥ 1.2.

A total of 2,070 transcripts were differentially expressed between non-WB and WB samples (Figure 1). Among the broilers slaughtered at the age of 6 weeks, 509 transcripts were up-regulated, and 401 transcripts were down-regulated in WB in comparison with non-WB samples. As for 7wk, 547 transcripts were up-regulated whereas 648 transcripts were down-regulated upon development of WB myopathy. Top 10 most up-regulated and down-regulated transcripts of the 6wk and 7wk samples are shown in Tables 1, 2, respectively. As illustrated in Venn diagram (Figure 1), only 35 out of 2,070 DE transcripts were identified in both 6 and 7 week comparisons with comparable FC (Table 3), except for the transcript encoding four and a half LIM domains 5 (*FHL5*) which showed 1.6-fold decrease in 6wk WB but 1.4-fold increase in 7wk WB compared to the expression of their non-WB counterparts. The complete DE transcripts lists are available in Supplementary Table 2.

**TABLE 1:** Top 10 most differentially expressed transcripts associated with wooden breast in 6-week-old broilers.

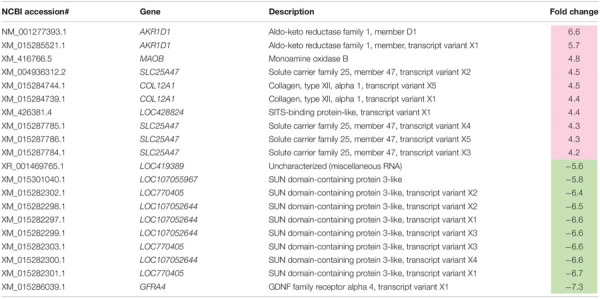

*Positive fold change, highlighted in red, and negative fold change, highlighted in green, indicate increase and decrease, respectively, in transcript abundance in wooden breast muscle samples collected from commercial broilers at 6 weeks of age.*

**TABLE 2:** Top 10 most differentially expressed transcripts associated with wooden breast in 7-week-old broilers.

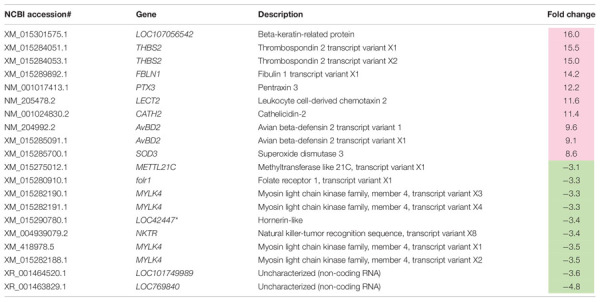

*A total of 36 differentially expressed transcripts were annotated as transcript variants of hornerin-like with fold change ranging from −3.4 to −4.1. Positive fold change, highlighted in red, and negative fold change, highlighted in green, indicate increase and decrease, respectively, in transcript abundance in wooden breast muscle samples collected from commercial broilers at 7 weeks of age.

**TABLE 3:** Differentially expressed transcripts associated with development of wooden breast myopathies identified in skeletal muscles of both 6-week-old and 7-week-old commercial broilers.

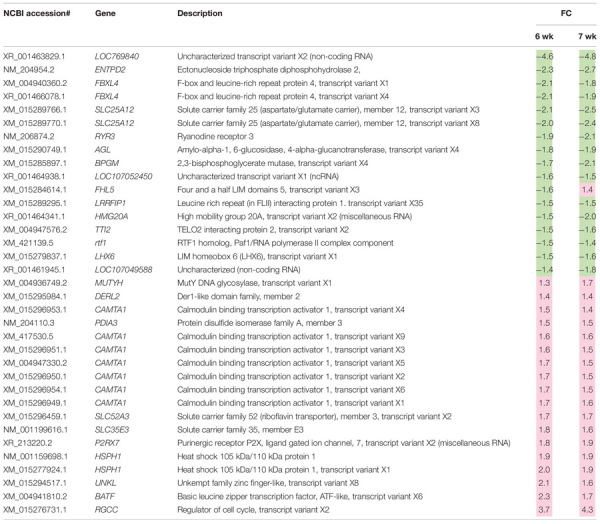

*FC, fold change; wk, week. Negative FC, highlighted in green, and positive FC, highlighted in red, indicate decrease and increase, respectively, in transcript abundance in wooden breast muscle samples collected from commercial broilers at 6 or 7 weeks of age.*

**FIGURE 2:**
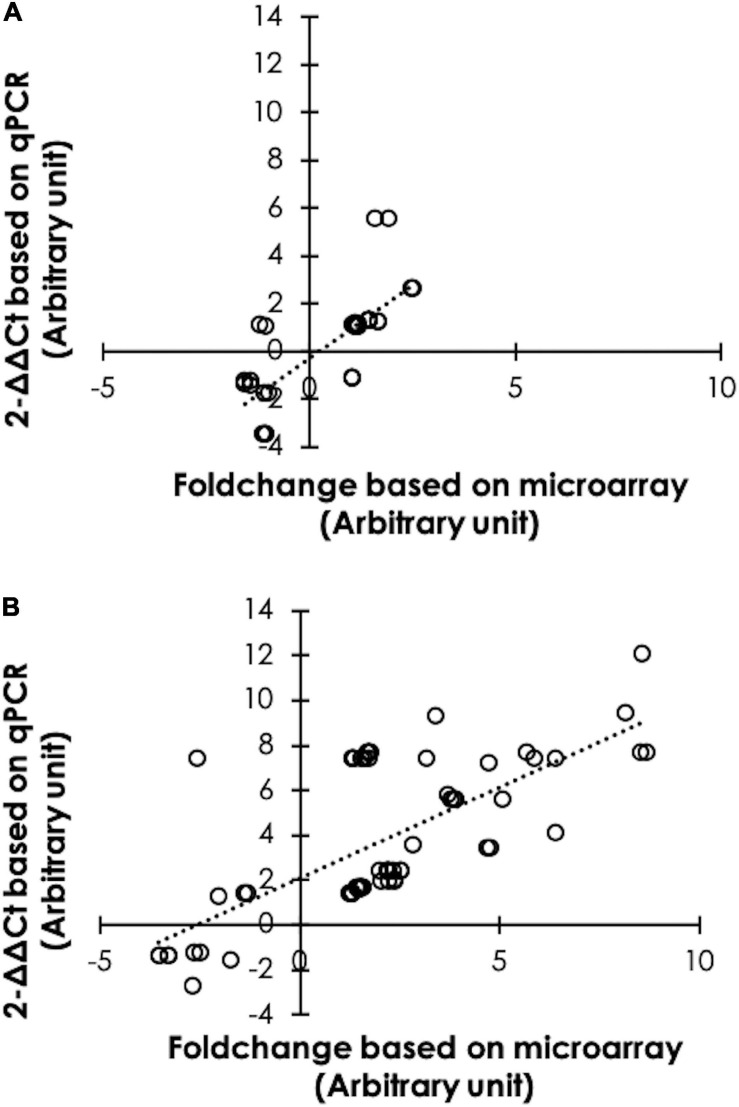
Confirmation of differential expression. Scatter plots illustrate the comparisons between fold changes of 40 differential expressed (DE) transcripts selected from 6-week-old broilers **(A)**, and of 88 DE transcripts from 7-week-old broilers **(B)** obtained from microarray and quantitative real-time polymerase chain reaction (qPCR). The results indicated that 36 (accounted for 87.5%) and 84 (accounted for 95.5%) counterparts show comparable results between microarray and qPCR.

Differential expression from microarray analysis was further confirmed by using a qPCR (Figure 2). As for 6 wk samples, 40 DE transcripts were tested. The findings indicated that 35 counterparts, accounting for 87.5% of the total, exhibited similar trends (Figure 2A). For the 7wk broilers, 84 out of 88 FC values, accounting for 95.5%, obtained from qPCR showed similar trend with the microarray results (Figure 2B).

### Functional and Pathway Analysis

**FIGURE 3:**
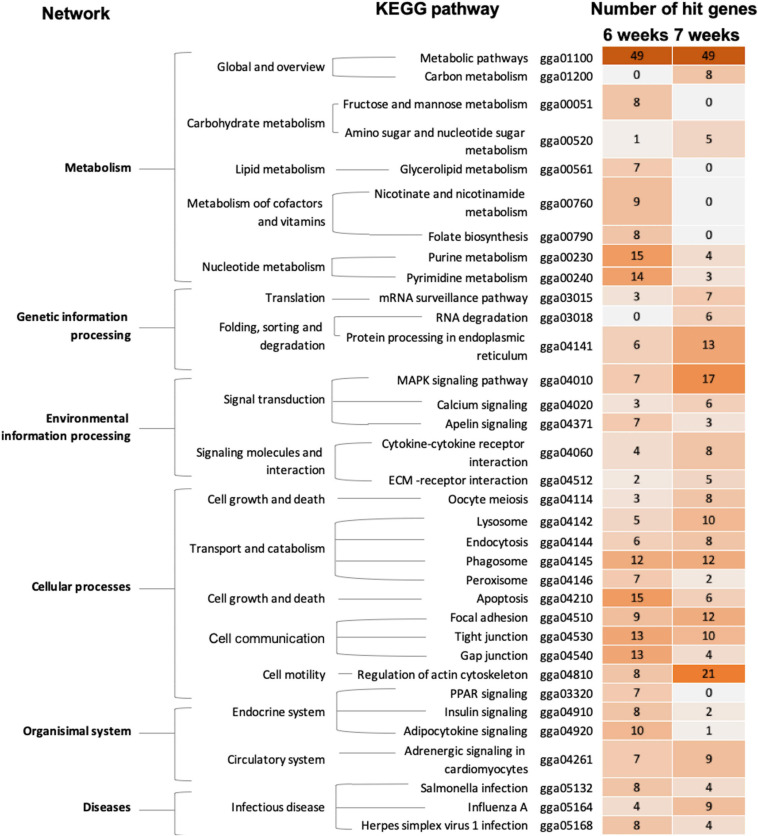
The enriched biological pathways associated with wooden breast (WB) myopathy in 6-week-old and 7-week-old broilers. Heatmap depicts the enriched altered biological pathways, which were further grouped into biological networks, based on KEGG database. The darker color indicated more numbers of differentially expressed genes mapped into the pathway.

Based on pathway analysis (Figure 3), alterations of metabolic pathways, cellular processes, particular transport and catabolism through phagosome, as well as cell communication *via* tight junction and FA, were highlighted in both 6wk and 7wk broilers. In 6wk group, metabolisms of purine and pyrimidine together with apoptosis, communication among cells through tight junction, gap junction, and FA were enriched altered pathways. Regulation of actin cytoskeleton signaling, mitogen−activated protein kinase (MAPK) signaling, protein processing in endoplasmic reticulum, FA, phagosome, and lysosome were enriched in the 7wk group.

### Changes in Absolute Expression of Key Genes in Focal Adhesion Signaling Pathway

**FIGURE 4:**
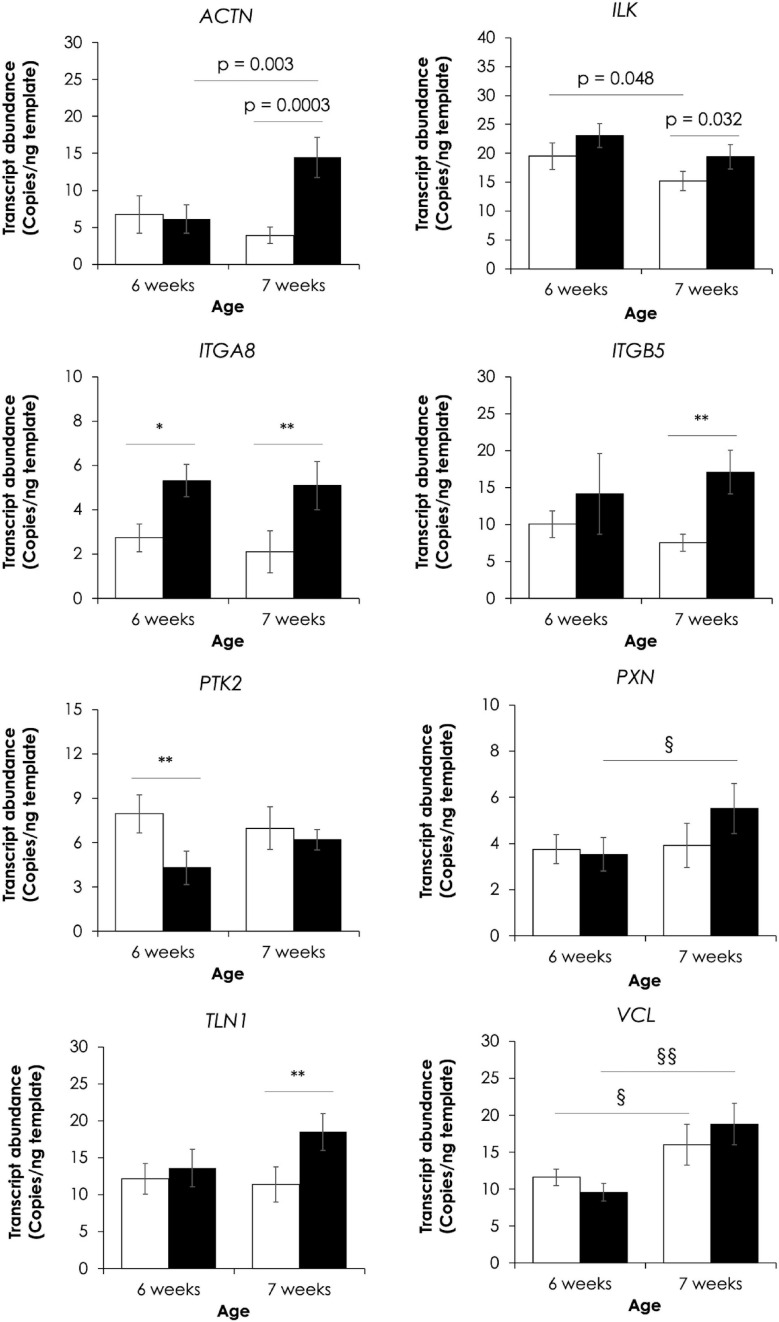
Effects of wooden breast myopathy on absolute expression of eight genes in focal adhesion signaling. The genes include actinin-1 (*ACTN1*), integrin-linked kinase (*ILK*), integrin subunit alpha 8 (*ITGA8)*, integrin subunit beta 5 (*ITGB5*), protein tyrosine kinase 2 (*PTK2*), paxillin (*PXN*), talin 1 (*TLN1*), and vinculin (*VCL*). Bars and error bars depict average and standard error in copies per nanogram cDNA template. *p < 0.05, **p < 0.01 between non-WB and WB samples. ^§^*p* < 0.05, ^§§^*p* < 0.01 between different ages.

Absolute expressions of eight genes in FA signaling were further elucidated using ddPCR (Figure 4). For 6 wk, only two genes, including integrin subunit alpha 8 (*ITGA8*, FC = 2.0) and protein tyrosine kinase 2 (*PTK2*, FC = −2.0), were found differentially expressed upon development of WB abnormality (*p* < 0.05). On the other hand, increases in actinin-1 (*ACTN1*, FC = 3.7), integrin-linked kinase (*ILK*, FC = 1.3), *ITGA8* (FC = 2.4), integrin beta subunit 5 (*ITGB5*, FC = 2.3), and talin 1 (*TLN1*, FC = 1.6) were observed in 7wk WB muscles in respect to their non-WB counterparts (*p* < 0.05). In addition, effects of different slaughtered ages on absolute transcript levels in WB muscles were detected for *ACTN1*, *PXN*, and *VCL* whereas such effects in non-WB muscles were observed for *ILK*, and *VCL* (*p* < 0.05).

## Discussion

### Six-Week-Old Broilers

Metabolic processes of carbohydrates, glycerolipids, cofactors and vitamins, and nucleotides (i.e., purine and pyrimidine) were identified as enriched pathways associated with the development of WB in 6wk broilers (Table 4 and Figure 3). These findings are in agreement with those obtained in previous studies performed on WB muscles (Mutryn et al., 2015; Abasht et al., 2016; Zambonelli et al., 2016). In detail, among the DE transcripts listed in the pathways (Supplementary Table 3), aldo-keto reductase (ARK), family 1 member D1 (*AKR1D1*, FC = 6.6) was also listed as the top up-regulated genes in 6wk WB if compared to their unaffected counterparts. Another gene encoding ARK family 1 member 10 (*ARK1B10*) was also increased for 2.6-fold. The ARK superfamily catalyzes oxidation-reduction reactions to detoxify aldehydes and ketones generated during metabolisms or in response to stresses (Huang et al., 2016). Increased expressions of those enzymes could be a consequence of the muscle fibers encountering stressful conditions associated with WB myopathy. In addition, an overexpression of *ARK1B10* was shown to elevate β-catenin, an activator of canonical Wnt signaling, in breast cancer cell lines (Qu et al., 2019). Within this context, the findings of the present study further support the hypothesis formulated by Phillips et al. (2020) in which, in the presence of WB myopathy, satellite cells could lose their capability of proliferation and differentiation due in part to an excessive activation of Wnt signaling pathway that, ultimately, results in the disruption of the muscle repair process.

**TABLE 4:** Top 10 enriched biological pathways associated with development of wooden breast myopathies in 6-week-old and 7-week-old commercial broilers.

Age	Pathway ID	KEGG pathways	Numbers of hit genes
6 weeks	gga01100	Metabolic pathways	49
	gga04210	Apoptosis	15
	gga00230	Purine metabolism	15
	gga00240	Pyrimidine metabolism	14
	gga04540	Gap junction	13
	gga04530	Tight junction	13
	gga04145	Phagosome	12
	gga04920	Adipocytokine signaling pathway	10
	gga00760	Nicotinate and nicotinamide metabolism	9
	gga04510	Focal adhesion	9
7 weeks	gga01100	Metabolic pathways	49
	gga04810	Regulation of actin cytoskeleton	21
	gga04010	MAPK signaling pathway	17
	gga04141	Protein processing in endoplasmic reticulum	13
	gga04510	Focal adhesion	12
	gga04145	Phagosome	12
	gga04142	Lysosome	10
	gga04530	Tight junction	10
	gga04261	Adrenergic signaling in cardiomyocytes	9
	gga05164	Influenza A	9

Grouped in the category of endocrine signaling, alteration of insulin, peroxisome-proliferator-activated receptors (PPAR), and adipocytokine signaling were more pronounced in the 6wk broilers compared to 7wk samples (Figure 3). Dysfunction of those signaling pathways caused by, or in association with, excess adipose tissues and insulin resistance leads to negative metabolic effects (Mashek et al., 2007; Contreras et al., 2019). Lake and Abasht (2020) addressed the resemblance between metabolic complications and muscle structural features of WB myopathy in broilers and that of diabetes in smooth and cardiac muscles of mammals. In this study (Supplementary Table 3), expressions of long-chain acyl-CoA ligase 1 (*ACSL1*), encoding the enzyme catalyzing conversion of fatty acid to acyl-CoA, and solute carrier family 27 member 2 (*SLC27A2*), which encodes fatty acid transporter protein (FATP), were reduced in WB at both ages for 1.7 and 1.9-fold, respectively, compared with those of non-WB samples. These results indicated a decreased β-oxidation likely associated with the accumulation of long-chain and very-long chain fatty acids (Mashek et al., 2007) in WB broilers which were well in agreement with the compromised glucose and lipid metabolisms, impaired energy production and fat deposition observed in the affected muscle (Abasht et al., 2016; Lake et al., 2019; Malila et al., 2019; Papah and Abasht, 2019; Lake and Abasht, 2020). Such impairment was further emphasized by an increased transcript abundance of the gene encoding 5′-AMP-activated protein kinase (AMPK) subunit beta-1 (*PRKAB1*, FC = 1.7), the conserved domain which acts as a positive regulator of AMPK complex (Supplementary Table 3).

Apart from metabolism of biochemical molecules, pathway enrichment analysis also reveals the manifestation of apoptosis in 6wk WB-affected broilers (Table 4). An impaired programmed cell death in the birds exhibiting growth-related myopathies was previously addressed (Malila et al., 2020; Phillips et al., 2020). In the present study, increased transcriptional levels of cathepsin B (*CTSB*, FC = 2.0), α-tubulin 1C (*TUBA1C*, FC = 1.7) along with a decrease in serine/threonine protein kinase B (*AKT3*, FC = −1.3) were observed in 6 week WB samples. The up-regulation of *CTSB* was previously associated with an enhanced activity of cathepsin B and L in WB breasts measured 12 h postmortem (Hasegawa et al., 2020). In addition, Mizunoe et al. (2020) recently reported that an overexpressed *CTSB* caused dysfunction of lipid metabolism in obese white adipose tissue potentially through a chronic state of low-grade inflammation, which was relevant to the condition of WB myopathy. Tubulins, dynamic polymers of microtubules, play important roles in various cellular functions through continual cytoskeletal reorganization in response to developmental and environmental cues (Ambriz et al., 2018). Chemical suppression of microtubules dynamic affected signaling processes that ultimately interfered apoptosis (Mollinedo and Gajate, 2003). It is interesting that Lake et al. (2019) reported an almost 1.7-fold decreased expression of gene encoding α-tubulin 8b in 2-week-old chicks that later (at 7 weeks of age) exhibited WB abnormality in comparison with unaffected broilers (Lake et al., 2019). The opposite directional changes of *TUBA* expression observed in a previous as well as in the current study might be an implication of a difference in the microenvironment at the different stage of WB myopathy (Ambriz et al., 2018). In addition, knockdown of *AKT3* was shown to limit cell proliferation and attenuated mammalian target of rapamycin (mTOR) signaling in the brain of the mouse (Easton et al., 2005), suggesting that the mediated apoptosis in 6wk WB muscle might be attributed to a modified mTOR signaling.

The other enriched altered pathways in the 6wk broilers included gap junction, tight junction and FA (Table 4), which are the adhesive structures of cell adhesion molecules in the cell surface that regulate of cell polarity and cell-cell communication (Rao and Wu, 2021). Dysregulation of those cell adhesions together with extracellular matrix (ECM) promotes pathological conditions associated with several diseases in humans (Rao and Wu, 2021). The DE transcripts enlisted in those pathways (Supplementary Table 3) include *TUBA1C* (FC = 1.7), Platelet-derived growth factor (PDGF) subunit A (*PDGFA*, FC = −1.4), and subunit B (*PDGFB*, FC = −1.5), serine/threonine-protein phosphatase PP1 catalytic subunit (*LOC100858156*, FC = −1.4), *AKT3* (FC = −1.3) and laminin gamma 1 (*LAMC1*, FC = −1.3). PDGF is considered one of the growth factors regulating cell proliferation, differentiation and angiogenesis through the binding with its receptor located on cell surface (Okonkwo et al., 2020). Up-regulations of its PDGF receptor (PDGFR) at gene level were addressed to be associated with increased fibrosis in WB fillets compared with non-WB counterparts (Papah et al., 2018; Pampouille et al., 2019). Although no differences in transcriptional level of PDGFR were identified in the 6wk WB broilers, we observed, in the affected muscles, a reduction of both *PDGFA* and *PDGFB*, which in mice has been reported to disrupt the wound repair process potentially through the perturbation of angiogenesis as previously observed in diabetic skin wound healing (Beer et al., 1997; Okonkwo et al., 2020). Furthermore, reduced expression of *LAMC1* was previously reported in patients with abnormal coronary atherogenesis (Hebbel et al., 2020), suggesting maladaptive activities of the cells when *LAMC1* expression was low. The importance of *LAMC1* in WB myopathy was recently evidenced in the study of Bordini et al. (2021) as it was defined as one of the hub genes interconnecting the DE gene network in WB and WS affected broilers.

Overall, the intensive perturbations of metabolic processes observed in 6wk broilers, with special reference to glucose and lipid metabolisms, are pronounced in WB broilers along with the convergence of cell-cell communication that regulates various cellular activities, including the processes of muscle repair.

### Seven-Week-Old Broilers

Metabolic pathway was one of the top enriched altered pathways observed in 7wk broilers (Table 4). Considering the DE transcripts listed in the metabolic pathways (Supplementary Table 4), however, the DE transcripts in such pathway were different from those observed in 6 week birds. Indeed, for 7wk samples, a number of those DE transcripts was involved in oxidative phosphorylation, and metabolisms of amino acids (argininosuccinate synthase, *ASS1*, FC = 2.5), amino sugars (glutamine-fructose-6-phosphate transaminase, *GFPT1*, FC = 2.0; sialic acid synthase, *NANS*, FC = 1.7; hexosaminidase, *HEXB*, FC = 2.1), proteoglycans (alpha-1,2-mannosyltransferase, *ALG11*, FC = −2.7; *ALG12*, FC = 1.9; alpha-1,6-mannosylglycoprotein 6-beta-N-acetylglucosaminyltransferase A, *MGAT5*, FC = 1.3; carbohydrate 4-sulfotransferase 9, *CHST9*, FC = 1.9), and precursor molecules required for biosynthesis and function of lysosomes (acid ceramidase, *ASAH1*, FC = 1.6; adenosine triphosphatase, *ENTPD2*, FC = −2.7). Regarding the biological roles of those DE transcripts, the profound disruption in the molecular events responsible for cell communication, cell transportation, and catalytic processes in the 7wk WB-affected samples is anticipated. The speculation is supported by the presence of phagosome, lysosome, and FA, regulation of actin cytoskeleton, apoptosis, endocytosis and ECM-receptor interaction in the enriched altered pathways (Table 4 and Figure 3). In addition, mitogen-activated protein kinase (MAPK) signaling, protein processing in endoplasmic reticulum, and cytokine-cytokine receptor interaction were among those enriched pathways.

The molecular events in the 7wk affected broilers were corresponded with the perturbed processes of muscle regeneration (Domingues-Faria et al., 2016). Expression of C-C motif chemokine ligand 19 (*CCL19*) was increased for 3.7-fold in 7wk WB-affected broilers if compared to their non-WB counterpart, corresponding with the findings of Hughes et al. (2020) in which up-regulated *CCL19* was identified in human skins in response to inflammation. CCL19 binds to C-C chemokine receptor (CCR) type 7 (*CCR7*), playing roles in trafficking of immune cells (Yoshida et al., 1997). Cathepsin S (*CTSS*), another lysosomal protease participating in degradation of ECM and inflammatory processes, was increased for more than 2.4-fold in 7wk WB-affected broilers. Increased expression of *CTSS* was demonstrated in response to the secreted pro-inflammatory cytokines (Fu et al., 2020). Elevated abundances of *CC7* (FC = 2.3), *CCR5* (FC = 2.1), and tumor necrosis factor receptor superfamily member 18 (*TNFRSF18*, FC = 2.4), and neutrophil cytosolic factor 4 (*NCF4*, FC = 2.0) were observed in the affected birds. Overall, these findings supported the recruitment of immune cells to the affected breast muscle.

It is evident that the main components of cytoskeleton were manifested under WB condition. Actin gamma 1 (*ACTG1*, FC = 1.8), beta-keratin related protein (*LOC10705654*2, FC = 16.0), α-tubulin 3 (*LOC416695*, FC = 3.0), and α-tubulin 4a (*TUBA4A*, FC = −1.5), the consisting proteins in those structures, were differentially expressed between WB and non-WB samples in the 7 week broilers (Supplementary Table 2). Muscle RAS oncogene homolog (*MRAS*), a member of small G proteins responsible for signal transduction and reorganization of actin cytoskeleton (Matsumoto et al., 1997; Kimmelman et al., 2002), was increased for 3.6-fold in the abnormal samples. Decreased transcript abundances of fibroblast growth factor 16 (*FGF16*, FC = −2.7) and son of sevenless (*SOS2*, FC = −1.5), Ras-guanine exchange factor, were detected in the 7wk affected broilers. As observed in the previous study of Sofronescu et al. (2010), activation of *FGF16* at transcriptional level was essential for cardiac growth and postnatal development of human and mice while Guittard et al. (2015) reported limited peripheral T-cell migration, hence impairing immune response when *SOS2* was absent.

Regarding the altered processes of cell communication (Table 4), transcript abundance of the gene encoding for one of the epidermal growth factor receptors (EGFR) belonging to the ErbB family of receptor tyrosine kinases, *ERBB2*, was decreased for 1.5-fold in respect to WB myopathy. This receptor is involved in the stabilization of peripheral microtubules in cell adhesion, triggering a rich network of signaling pathways (Yarden and Sliwkowski, 2001). Fibronectin 1 (*FN1*) was up-regulated for 2.7-fold in WB-affected 7wk broilers. Widely distributed in inflammatory lesions, fibronectins, a group of fibroblast surface glycoproteins, generally binds to ECM proteins, were also found to play a major role during wound healing (Grinnell et al., 1981). An extensive localization of FN1 at endomysial and perimysial levels was found in WB coupled with an increased content of this glycoprotein (Praud et al., 2020). In addition, grouped in phagosome, FA (Table 4), and ECM receptor interaction (Supplementary Table 4), thrombospondin 2 (*THBS2*), disulfide-linked homotrimeric glycoprotein, was highlighted as one of the most highly increased transcripts (FC = 15) in 7wk WB-affected broilers if compared to their non-WB counterparts (Table 2). This transient matrix glycoprotein inhibits microvascular endothelial cells proliferation thus resulting in impaired angiogenetic processes and limiting vascularization (Zhang et al., 2020). Intriguingly, these features overlap with the microscopic traits observed in WB affected muscles (Abasht et al., 2021).

A profound impairment of muscle regeneration in 7wk WB-affected broilers was further supported by the expression levels of genes responsible for protein processing within the endoplasmic reticulum (ER, Table 4 and Figure 3). Indeed, increased expressions of genes encoding for several chaperone proteins, including heat shock protein (HSP) 110 kDa (*HSPA4L*, FC = 1.5) and 70 kDa (*HSPA5*, FC = 1.7) were found. In particular, a 1.5-fold increase in protein disulfide-isomerase A3 (*PDAI3*) was found in WB muscles (Supplementary Table 4). This multifunctional chaperone is originally located within ER but can escape to cell surface and interacts with integrins. Wang et al. (2020) recently demonstrated that a chemical inhibition of PDIA3 reduces myoblast differentiation in injured skeletal muscle, ultimately impairing muscle regeneration. An activation of ER-associated degradation, the process to eliminate misfolded proteins retained within the ER lumen through the proteasome to restore normal ER function, might be speculated. Under severe stress condition in which ER function was not recovered, the affected cells were entered programmed cell death.

The impaired muscle regeneration in 7wk affected broilers perfectly matches with the altered muscular structure observed associated to WB condition (Velleman, 2020). In this study, a decreased transcript abundance of myopalladin (*MYPN*, FC = −1.4) and myomesin 1 (*MYOM1*, FC = −1.7) together with increased expressions of several genes encoding collagens (*COL1A1*, FC = 2.1, *COL8A1*, FC = 2.0, *COL9A2*, FC = 2.2, *LOC101750377*, FC = 2.1) were found in 7wk affected broilers in comparison with non-WB ones (Supplementary Table 2). MYPN tethers nebulin to actinin at Z-line, maintaining sarcomeric integrity (Bang et al., 2001) whereas MYOM1, a major component of myofibrillar M band expressed in almost all striated muscles, stabilizes the three-dimensional structure of the thick filaments by linking together myosin, titin, and light meromyosin (Agarkova and Perriard, 2005). Overall, gene expression is in agreement with the severe disruption of muscular structure coupled with accumulation of collagen observed in WB muscle (Velleman, 2020).

Collectively, differential expression of those genes underlined an extensive molecular alteration among the biological networks playing roles in response to severe muscle damage in the 7wk WB-affected broilers.

### Transcriptional Modification of Focal Adhesion Signaling

**FIGURE 5:**
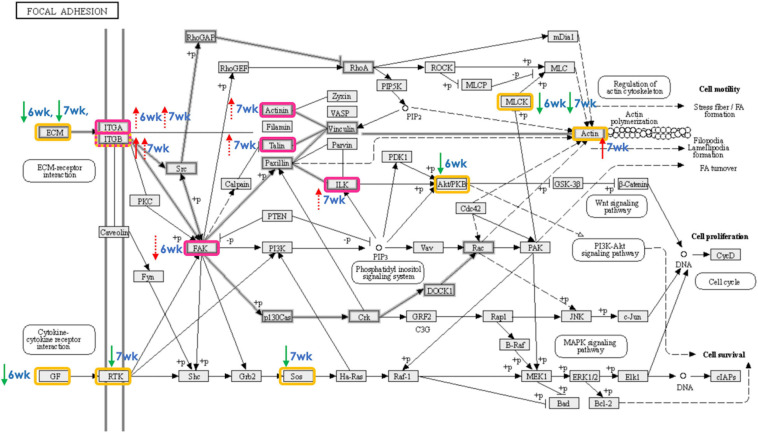
Focal adhesion signaling pathway as affected by development of wooden breast (WB) in 6-week-old and 7-week-old broilers. The proteins highlighted in yellow and pink boxes exhibited differential expression, as detected using microarray and droplet digital polymerase chain reaction, respectively, in skeletal muscle of broilers affected WB myopathy in comparison with their non-defective counterparts. The red and green arrows (solid = microarray, dashed = ddPCR) indicate the increased and decreased expression, respectively, of the gene-encoded protein in WB relative to non-WB of 6-week-old (6wk) or 7-week-old (7wk) broilers.

The main enriched biological pathways associated with the development of WB myopathy in 6wk and 7wk was somewhat different. The molecular disruption in 6wk was more toward metabolic pathways for glucose and lipids whereas an aberrant muscle repair process has a more relevant importance in 7wk samples. Aside from that, the presence of FA signaling was similarly found in both groups, suggesting the relevance of this process in WB development. Here, we further compared absolute transcript abundances of the eight protein components of FA signaling between non-WB and WB samples within each slaughter age using ddPCR (Figure 4). The ddPCR technique permits the transcriptional quantification with good precision and sensitivity (Taylor et al., 2017). Therefore, the results could extend the comparative analysis of microarray which normally showed some limitation based on fixed dynamic range of fluorescence (Sharov et al., 2004). Increases or decreases in transcript abundance detected by both microarray and ddPCR studies in 6wk and 7wk were summarized in Figure 5.

Focal adhesion, the integrins-mediated contact sites between cell and ECM, acts as the linking points that mediates mechanical and biochemical signaling from the ECM (Turner, 2000; Wozniak et al., 2004; Huveneers and Danen, 2009). The reorganization of actin cytoskeletal structure regulated by FA signal leads to a series of intracellular events in response to the extracellular initiator. The crosstalk is crucial for controlling several cellular mechanisms, from fundamental development through recovery from any injuries or infection, and, in turn, maintaining tissue homeostasis, thus organism’s survival (Zaidel-Bar and Geiger, 2010). A disrupted FA signaling accompanied by defective ECM and insufficient angiogenesis, which results in poor tissue oxygenation (condition consistently observed within the breast muscles of commercial broilers exhibiting growth-related myopathies), was associated with the impaired wound healing that led to an aberrant deposition of ECM molecules (e.g., collagen and decorin) and ultimately resulting in fibrosis (Koivisto et al., 2014).

Corresponded with the current results, up-regulated *ITGA8* was previously reported in broiler breast muscle affected by WS abnormality (Marchesi et al., 2019), and in specimen of 90 patients with hepatic fibrosis (Nishimichi et al., 2021). In addition, the same authors found that *ITGA8* was minimally expressed in healthy liver, underlying its relevance with liver fibrosis. Induced *ITGB5* was found to directly interact with β-catenin, facilitating Wnt/β-catenin activity in hepatocellular carcinoma (Lin et al., 2018). Deletion of *FAK* in mouse liver accelerated liver regeneration through the increased TNFα as well as the activation of apoptosis (Shang et al., 2016). Decreased *PTK2*, gene encoding for FAK, found in 6wk WB broilers may be the result of an adaptive response through FAK-mediated FA to accelerate the removal of damaged cells through apoptosis, and partly mitigate the development of fibrosis within the muscle (Lagares et al., 2012; Velleman, 2020).

Reduced transcript abundances of genes encoding for ECM components (*LAMC1* in 6wk, *FN1* in 7wk), and growth factors (*PDGFA* in 6wk) were identified, based on the current microarray study, in the WB-affected muscles. In contrast, the majority of the genes encoding for FA components tested exhibited increased expression levels. Those genes encode intracellular proteins that mediate integrin binding to actin microfilament, maintain stability of the integrin adhesion, and sense matrix rigidity (Klapholz and Brown, 2017). Most of the transcriptional changes in this pathway were detected in 7wk broilers. The findings suggested an altered crosstalk between immune cell and muscle cells may be hypothesized as a consequence of the chronic inflammatory processes, leading to failure in muscle regeneration (Domingues-Faria et al., 2016).

In conclusion, our findings were congruent with the proposed model of ECM disorders in the pathogenesis of growth-related myopathies in broilers (Bordini et al., 2021). The present study further provides an observation on the alteration of ECM-mediated FA signaling. Such impairment exerted the deleterious effects on immune response and muscle regeneration process at a greater extent in 7wk broilers. Furthermore, molecular perturbation pattern associated with WB development differed between 6wk and 7wk birds. In 6wk broilers, the metabolic processes of glucose and lipid, in particular, were deviated between WB and non-WB group. On the other hand, the importance of impaired muscle regeneration processes was evident in the 7wk affected broilers. Whether the altered FA signaling was a cause or effects of those dysregulated biological pathways remained to be further investigated.

## Data Availability Statement

The datasets presented in this study can be found in online repositories. The names of the repository/repositories and accession number(s) can be found below: https://www.ncbi.nlm.nih.gov/, GPL24307; https://www.ncbi.nlm.nih.gov/, GSE107362.

## Ethics Statement

Ethical review and approval was not required for the animal study because all samples used in this study were purchased in the form of whole carcasses from the commercial processing plant without any experimental treatments subjected to the living animals. Thereby, the ethical approval was exempted.

## Author Contributions

YM, WR, and WV conceived and designed the experiments as well as acquired the funding. YM, TU, YS, SA, and KT performed the experiments and analyzed the data. TU performed bioinformatics. KT performed ddPCR. YM drafted the manuscript with revisions provided by TU, WR, MP, and FS. All authors read and approved the final manuscript.

## Conflict of Interest

The authors declare that the research was conducted in the absence of any commercial or financial relationships that could be construed as a potential conflict of interest.

## Footnotes

1 https://www.ncbi.nlm.nih.gov/tools/primer-blast/
